# Supplementary material for: Enhanced Recovery after Surgery (ERAS) Implementation and Barriers among Healthcare Providers in France: A Cross-Sectional Study
Source: Healthcare (Basel). 2024 Feb 8;12(4):436. doi: 10.3390/healthcare12040436 (PMC10887527; doi:10.3390/healthcare12040436)
Supplement: Supplementary file 1 [file healthcare-12-00436-s001.zip › healthcare-2758669-supplementary.pdf]

Enhanced Recovery After Surgery is little practiced due to environmental context and resources as well as knowledge barriers: a cross-sectional study.

Supplementary Materials

Augustin Clet, PT, MSc (1,2) ; Marin Guy, PT, MSc (3) ; Jean-François Muir, MD, Prof (1,2,4) ; Antoine Cuvelier, MD, Prof (2,4) ; Francis-Edouard Gravier, PT, PhD (1,2) ; Tristan Bonnevie, PT, PhD (1,2) .

1. *Univ Rouen Normandie, Normandie Univ, GRHVN UR 3830, F-76000 Rouen, France*
2. *ADIR Association, Rouen University Hospital, Rouen, France*
3. *Centre Aquitain Du Dos, Hôpital Privé Saint-Martin, Pessac, France*
4. *Pulmonary, Thoracic Oncology and Respiratory Intensive Care Department, Rouen University Hospital, Rouen, France*

Supplementary S1: STROBE Statement—Checklist of items that should be included in reports of *cross-sectional studies*

	Item No	Recommendation	Page No
**Title and abstract**	1	(*a*) Indicate the study's design with a commonly used term in the title or the abstract	1
		(*b*) Provide in the abstract an informative and balanced summary of what was done and what was found	3-4
**Introduction**			
Background/rationale	2	Explain the scientific background and rationale for the investigation being reported	5
Objectives	3	State specific objectives, including any prespecified hypotheses	5-6
**Methods**			
Study design	4	Present key elements of study design early in the paper	6
Setting	5	Describe the setting, locations, and relevant dates, including periods of recruitment, exposure, follow-up, and data collection	6
Participants	6	(*a*) Give the eligibility criteria, and the sources and methods of selection of participants	7
Variables	7	Clearly define all outcomes, exposures, predictors, potential confounders, and effect modifiers. Give diagnostic criteria, if applicable	7
Data sources/ measurement	8*	For each variable of interest, give sources of data and details of methods of assessment (measurement). Describe comparability of assessment methods if there is more than one group	*6*
Bias	9	Describe any efforts to address potential sources of bias	6

Study size	10	Explain how the study size was arrived at	7
Quantitative variables	11	Explain how quantitative variables were handled in the analyses. If applicable, describe which groupings were chosen and why	8
Statistical methods	12	(*a*) Describe all statistical methods, including those used to control for confounding	8
		(*b*) Describe any methods used to examine subgroups and interactions	8
		(*c*) Explain how missing data were addressed	7
		(*d*) If applicable, describe analytical methods taking account of sampling strategy	/
		(*e*) Describe any sensitivity analyses	/
**Results**			
Participants	13*	(a) Report numbers of individuals at each stage of study—eg numbers potentially eligible, examined for eligibility, confirmed eligible, included in the study, completing follow-up, and analysed	8-9
		(b) Give reasons for non-participation at each stage	9
		(c) Consider use of a flow diagram	9
Descriptive data	14*	(a) Give characteristics of study participants (eg demographic, clinical, social) and information on exposures and potential confounders	9 ; 17-18
		(b) Indicate number of participants with missing data for each variable of interest	8
Outcome data	15*	Report numbers of outcome events or summary measures	9-11
Main results	16	(*a*) Give unadjusted estimates and, if applicable, confounder-adjusted estimates and their precision (eg, 95% confidence interval). Make clear which confounders were adjusted for and why they were included	9-11 ; 18-19
		(*b*) Report category boundaries when continuous variables were categorized	/
		(*c*) If relevant, consider translating estimates of relative risk into absolute risk for a meaningful time period	/
Other analyses	17	Report other analyses done—eg analyses of subgroups and interactions, and sensitivity analyses	10-11
**Discussion**			
Key results	18	Summarise key results with reference to study objectives	11
Limitations	19	Discuss limitations of the study, taking into account sources of potential bias or imprecision. Discuss both direction and magnitude of any potential bias	13-14
Interpretation	20	Give a cautious overall interpretation of results considering objectives, limitations, multiplicity of analyses, results from similar studies, and other relevant evidence	11-13
Generalisability	21	Discuss the generalisability (external validity) of the study results	11
**Other information**			
Funding	22	Give the source of funding and the role of the funders for the present study and, if applicable, for the original study on which the present article is based	/

*Give information separately for exposed and unexposed groups.

Note: An Explanation and Elaboration article discusses each checklist item and gives methodological background and published examples of transparent reporting. The STROBE checklist is best used in conjunction with this article (freely available on the Web sites of PLoS Medicine at http://www.plosmedicine.org/, Annals of Internal Medicine at http://www.annals.org/, and Epidemiology at http://www.epidem.com/). Information on the STROBE Initiative is available at www.strobe-statement.org.

Supplementary S2: English translation of the questionnaire

ERAS: What do healthcare providers know?

Administrative data

1. You are:
    - Men
    - Woman

Only one answer possible.

2. How old are you?

Only one answer possible.

3. What is your occupation?
    - Anesthesiologist
    - Dietician
    - General Physician
    - Medical Specialist
    - Nurse
    - Nurse anesthetist
    - Nursing Assistant
    - Occupational therapist
    - Operating room nurse
    - Physiotherapist
    - Private Nurse
    - Psychologist
    - Surgeon

Only one answer possible.

4. In which country were you trained?

Open text.

5. In what year did you graduate?

Only one answer possible.

6. What are your workplaces?

    - Health center
    - Hospital

- Private Hospital
- Private Practice
- Residential facility for dependent elderly people
- Others: (*Open text*)

Several possible answers.

7. In which department do you practice? (*Indicate the number of the department*)

Open text.

8. Have you ever heard of ERAS (Enhanced Rehabilitation After Surgery)?

- Yes, I practice it
- Yes, but I don't practice it
- No

Only one answer possible.

9. Is an ERAS protocol in place at your workplace?
   - Yes
   - No
   - I do not wish to comment.

Only one answer possible.

10. If yes, in which specialty(ies)/department(s)?

Open text.

I practice ERAS.

11. In which specialty/department do you practice as an ERAS practitioner?

Open text.

12. Of the following options, what do you identify as barriers to ERAS implementation?

- Changing care habits to adopt new practices.
- The absence of a financing nomenclature adapted to these patients.
- Lack of funding.
- The regular change of professionals within the department.
- The turnover in the installation of self-employed on the territory.
- The increase in the number of administrative tasks.
- Multidisciplinary teamwork.
- Poor city-hospital coordination.
- ERAS labels for healthcare facilities.
- None of the previous answers.

Several possible answers.

13. Do you identify other barriers to ERAS implementation, not mentioned above?

Open text.

I don’t practice ERAS

According to the Haute Autorité de Santé [French Health Authority]: "Enhanced Recovery After Surgery (ERAS), initially developed in the 1990s by the Danish team led by Professor Henrik Kehlet, is a comprehensive patient care approach that promotes the early recovery of their abilities after surgery. It is a multidisciplinary care approach aimed at reducing post-operative hospitalization time and post-operative complications. The key components of this care approach are primarily: a well-established care pathway with thorough patient information, minimally invasive surgery, reduced use of morphine, optimal pre and post-operative rehabilitation, early resumption of eating, and early mobilization."

14. Of the following options, what do you identify as barriers to ERAS practice?
    - I had never heard about it.
    - I have never seen a patient in a ERAS protocol.
    - I do not feel concerned by this type of care.
    - I do not wish to change my care habits.
    - I don’t have the time to ERAS patient.
    - I don’t have the means to ERAS patient.
    - The health care facility where I work does not have an ERAS protocol.
    - The health care facilities around my practice do not ERAS.
    - I have not been contacted by the health care facilities around my practice.
    - None of the previous answers.

Several possible answers.

15. Do you identify other barriers to ERAS practice, not mentioned above?

Open text.

16. Do you have any comments or suggestions?

Open text.

Supplementary S3. Table of characteristics of the pretest population for the questionnaire

Variable	n=9
**Age** (Years), *median (IQR)*	33 (*29-49*)
**Gender** n (%)	
Female	5 (55.6%)
**Profession,** n (%)	
Physiotherapist	4 (*44.4%*)
Nurse	1 (11.1%)
Occupational therapist	1 (11.1%)
Psychologist	1 (11.1%)

Nurse anesthetist	1 (11.1%)
Operating room nurse	1 (11.1%)
**Graduate country** n (%)	
France	9 (*100.0%*)
**Time from graduation** (Years), Median (IQR)	11 (5-25)
**Practice mode,** n (%)	
Employee	5 (55.6%)
Self-employed	4 (44.4%)

Supplementary S4: Theoretical Domains Framework : Support and definition

Theorical Domains of Framework	Content
**Knowledge**	An awareness of the existence of something
**Skills**	Ability or proficiency attained through practice
**Social and professional role/identity**	Behaviours and personal qualities displayed in a work or social setting
**Beliefs about capabilities**	Acceptance of the truth, reality, or validity about an ability, talent or facility that a person can put to constructive use
**Optimism**	Confidence that outcomes will be for the best and/ or goals will be met
**Beliefs about consequences**	Acceptance of the truth, reality, or validity about outcomes of a behaviour in a given situation
**Reinforcement**	Increasing the likelihood of a response through the existence of a dependent relationship between the stimulus and response
**Intentions**	A conscious decision to perform a behaviour or act in a particular way
**Goals**	Mental representation of desired outcomes to be achieved
**Memory, attention, and decision processes**	The ability to retain information, focus selectively and choose between multiple alternatives
**Environmental context and ressources**	Circumstances of a person's situation or environment that promote (or impede) the development of skills or behaviours

**Social influences**	Interpersonal processes that influence and individual to change their behaviour or thinking
**Emotion**	A complex reaction, drawing on experience, behaviour, and physiological components that allow an individual to attempt to deal with a significant event/issue
**Behavioral regulation**	Anything aimed at managing or changing objectively observed or measured actions

Supplementary S5: Allocation of domains of TDF from question Q12 to Q15

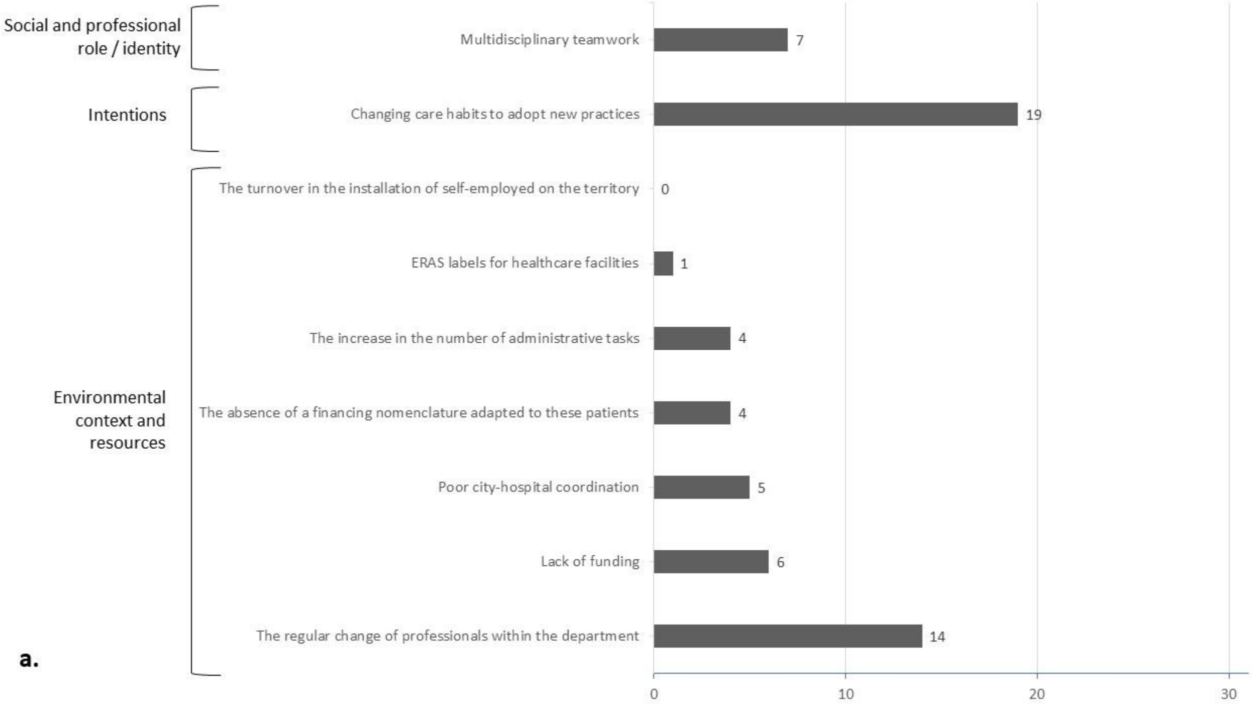

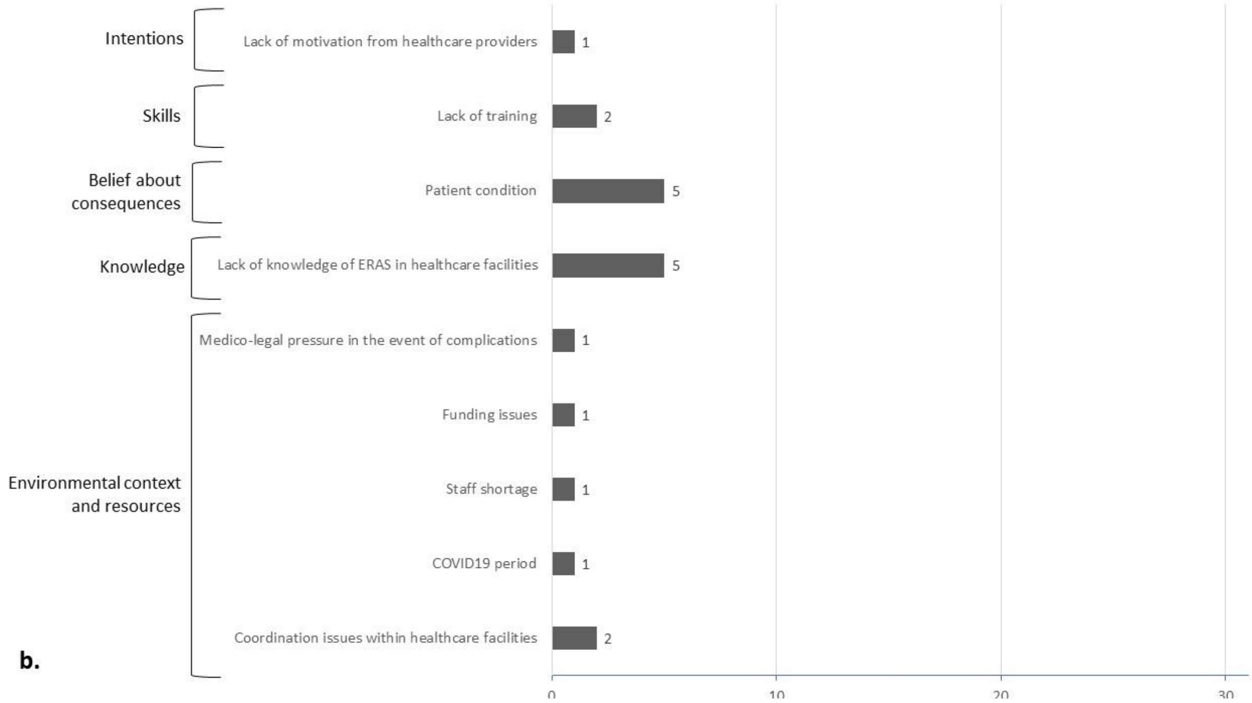
Intentions
Lack of motivation from healthcare providers
1
Skills
Lack of training
2
Belief about consequences
Patient condition
5
Knowledge
Lack of knowledge of ERAS in healthcare facilities
5
Environmental context and resources
Medico-legal pressure in the event of complications
1
Funding issues
1
Staff shortage
1
COVID19 period
1
Coordination issues within healthcare facilities
2
0
10
20
30

b.

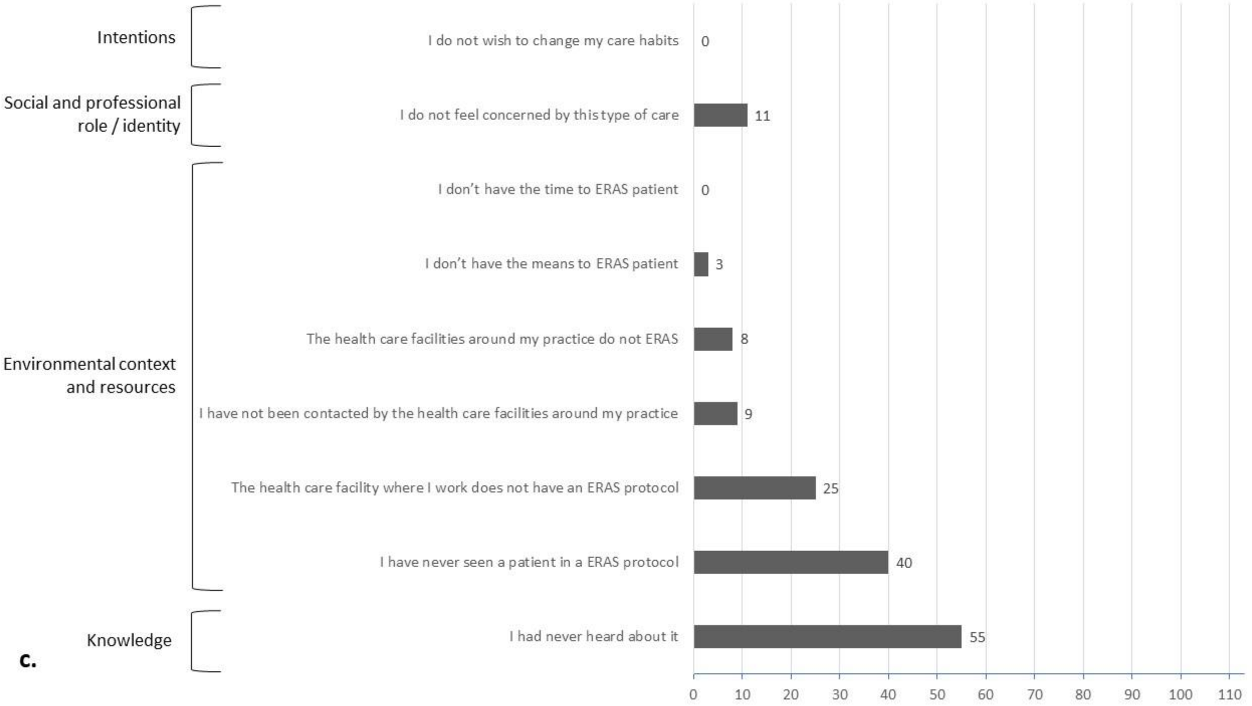
Intentions
I do not wish to change my care habits
0
Social and professional role / identity
I do not feel concerned by this type of care
11
Environmental context and resources
I don't have the time to ERAS patient
0
I don't have the means to ERAS patient
3
The health care facilities around my practice do not ERAS
8
I have not been contacted by the health care facilities around my practice
9
The health care facility where I work does not have an ERAS protocol
25
I have never seen a patient in a ERAS protocol
40
Knowledge
I had never heard about it
55
0 10 20 30 40 50 60 70 80 90 100 110

c.

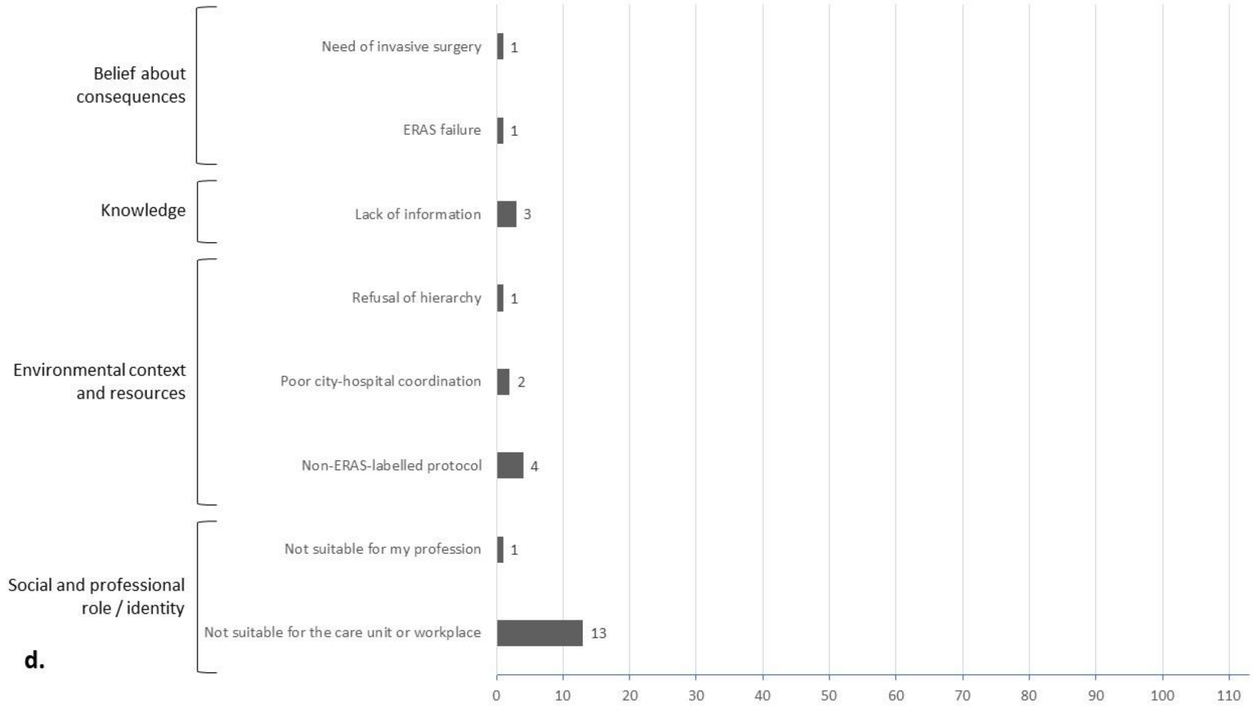

Figure S1: Results form Question Q12 to Q15.

Figure S1.a. responds to Question Q12, b. responds to Question Q13, c. responds to Question Q14 and d. responds to Question Q15. For Q13 and Q15, answers were bundled into similar topics expressed.
